# Icariin Prevents Diabetes-Induced Bone Loss in Rats by Reducing Blood Glucose and Suppressing Bone Turnover

**DOI:** 10.3390/molecules24101871

**Published:** 2019-05-15

**Authors:** Shanshan Qi, Jia He, Hongxing Zheng, Chen Chen, Shiqiang Lan

**Affiliations:** 1 College of Biological Science and Engineering, Shaanxi University of Technology, Hanzhong 723000, China; hejia@stu.snut.edu.cn (J.H.); lanshiqiang027@163.com (S.L.); 2 Chinese-German Joint Laboratory for Natural Product Research, Shaanxi University of Technology, Hanzhong 723000, China

**Keywords:** icariin, diabetic osteoporosis, bone turnover, bone histomorphometry

## Abstract

Diabetic Osteoporosis (DOP) is a common metabolic bone disease, characterized by decreased bone mineral density (BMD) and destruction of bone microstructure. It has been reported that icariin is beneficial for estrogen deficiency-induced osteoporosis, and alcohol-induced osteoporosis; whether icariin has protective effects on diabetes-induced osteoporosis has not been reported. In this study, a rat model of diabetic osteoporosis was established by streptozotocin injection, the bone protective effects and potential mechanism of icariin on diabetes-induced bone loss was observed. Thirty 8-week-old female Sprague Dawley rats were divided into control group (vehicle treatment), T1DM (diabetic) group and T1DM-icariin (ICA) group (diabetic rats treated with icariin), 10 rats in each group. The bone histomorphometry parameters, bone mineral density (BMD), serum bone turnover markers, and bone marrow adipogenesis were analyzed after 8 weeks of icariin administration. The results showed consumption of icariin at a doses of 100 mg kg^−1^ decreased blood glucose, and increased the BMD of diabetic rats. Icariin effectively decreased serum bone turnover marker levels, including CTX-1, ALP, TRACP 5b, osteocalcin, and PINP. Meanwhile, the bone histomorphometry parameters, the number of osteoclasts per bone perimeter were turned to be normal level, and the icariin treatment suppressed bone marrow adipogenesis. The runt-related transcription factor 2 (RUNX 2), as well as the osteoprotegerin (OPG)/receptor activator of nuclear factor-κ B ligand (RANKL) ratio in serum and bone tissues were increased significantly after icariin treatment in diabetic rats. All of the above indicate that oral administration of icariin can prevent diabetic osteoporosis; the effect is mainly related to its ability to reduce blood glucose, inhibit bone turnover and bone marrow adipogenesis, as well as up-regulate bone RUNX 2, and OPG expression.

## 1. Introduction

With the improvement of people’s living standards and changes in eating habit, the number of diabetic patients has been increasing. According to international diabetes federation (IDF), there are about 425 million diabetic patients (18–99 years old) worldwide at present, and the number will increase to 629 million by 2045 [1]. Diabetic osteoporosis (DOP) is a systemic metabolic bone disease affected by genetic and environmental factors, characterized by bone destruction and decreased bone density [2,3], it belongs to secondary osteoporosis (OP). There are many studies showing that the incidence of DOP is as high as 60% in diabetic patients [4,5]. DOP not only causes bone pain and fractures, but also seriously affects the life quality of patients, and brings a heavy economic burden to patients’ families and society. Therefore, the research and treatment of DOP have been paid more and more attention by researchers.

There are many differences between diabetic osteoporosis (DOP) and postmenopausal osteoporosis (PMOP). DOP is mainly related to the metabolic disorder caused by diabetes. Hyperglycemia can inhibit osteoblasts proliferation and promote osteoclasts differentiation, and decreased osteocalcin (OPG) expression, promote calcium loss, and decreased bone mineral density (BMD) [4]. Postmenopausal osteoporosis is mainly associated with increased bone resorption caused by decreased estrogen levels. The treatment of DOP should first control blood glucose; once diabetes is controlled, the indicators of diabetic osteoporosis can be improved, so it has a relatively short treatment course, whereas postmenopausal osteoporosis requires long-term treatment [6].

Although insulin has hypoglycemic effect, it can inhibit bone turnover; hormone therapy has side effects such as breast cancer, hypoglycemia, headache and flu-like symptoms [7,8,9]. The use of bone resorption inhibitors and bone formation promoters, such as bisphosphonates or raloxifene to treat DOP are costly and having adverse effects such as constipation and gastrointestinal irritation [10]. Thus, it is necessary to find a safe and effective natural product to prevent DOP.

**Figure 1:**
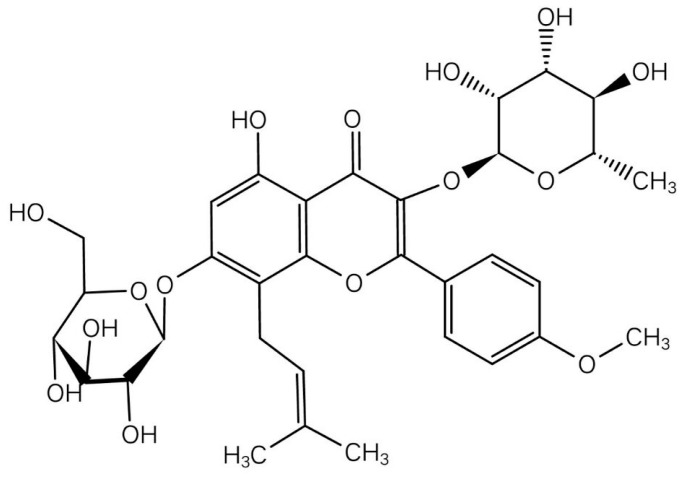
Chemical structure of icariin.

Icariin (ICA) is the main active ingredient of Chinese herbal medicine Epimedium (*Epimedium brevicornum Maxim*) and is a flavonoid extracted from Epimedium which has a long history of treating fractures in China, the chemical structure of icariin was indicated in Figure 1. Studies have reported that icariin can increase bone mineral density in ovariectomized rats [11,12,13], it also can restore bone structure and strength in a rat model of alcohol-induced osteopenia [14], as well as disuse osteoporosis in a rodent model [15]. However, whether icariin has protective effects on diabetes-induced osteoporosis has not been reported. It was reported that icariin also has other bioactivities such as improving dysfunction in spinal cord injury, neuroprotective effects, and anti-hepatotoxic activity [16]. Recently, it was reported that icariin has anti-diabetic effects, can inhibit adipocyte differentiation and improve insulin, blood glucose and lipid metabolism in diabetic mice, and improve diabetic complications such as retinopathy, and cognitive deficits [17,18,19]. We guess it maybe has a bone-protective effect on diabetes-induced osteoporosis. So in this study, we established a streptozotocin-induced diabetic osteoporosis rat model and administered icariin to investigate the protective effects of icariin on DOP and its possible mechanism. This study will provide new data for the adjuvant treatment of DOP, and provide some preliminary evidence for using icariin in DOP treatment.

## 2. Results

### 2.1. Icariin (ICA) Increased Bone Mineral Density of Diabetic Rats

**Figure 2:**
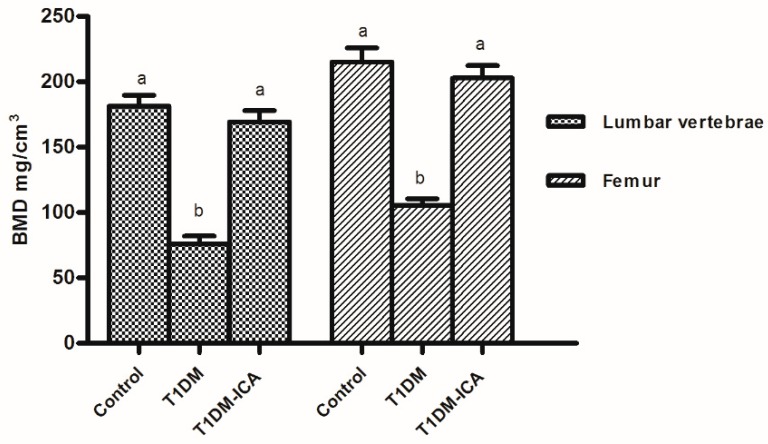
The lumbar vertebrae and femur bone mineral density (BMD) of rats in each group. Values are presented as means ± SD. Different letters were used to indicate statistically significance difference (*p* < 0.05).

Bone mineral density analysis results showed that the lumbar (L1–L4) and femoral bone mineral density (BMD) were decreased in diabetic rats (*p* < 0.05), which were recovered by ICA treatment (T1DM-ICA), there was significant difference in BMD between T1DM-ICA group and the T1DM group (*p* < 0.01) (Figure 2). These results suggest that ICA can increase BMD in diabetic rats.

### 2.2. ICA Decreased Blood Glucose and Serum Bone Turnover Markers

As Table 1 indicates, the blood glucose levels and serum bone turnover markers (ALP, CTX-1, osteocalcin, TRACP 5b, PIPN) in the T1DM group were higher than those in the control group (*p* < 0.01). After 8 weeks ICA administration, the serum blood glucose levels as well was the serum bone turnover markers were significantly decreased in T1DM-ICA group compared with the T1DM group (*p* < 0.01).

### 2.3. ICA Increased Serum Ca, P, OPG, and RUNX 2 and Decreased Serum RANKL

As shown in Table 2, serum RANKL in the T1DM group was higher than that in the control group (*p* < 0.01), which was decreased by ICA administration, and there was significant difference of serum RANKL level between T1DM-ICA and T1DM group (*p* < 0.01). There were decreased levels of serum calcium (Ca), phosphorus (P), OPG, RUNX 2, and OPG/RANKL ratio in the T1DM group compared with the control group (*p* < 0.01). After 8 weeks of ICA treatment, the levels of serum Ca, OPG, RANKL, and RUNX 2 were increased significantly, and there were significant differences of serum levels of Ca, RUNX2, OPG, and RANKL between T1DM-ICA and T1DM groups (*p* < 0.01).

### 2.4. Effects of ICA on Bone Morphology and Bone Histomorphometry Parameters

**Figure 3:**
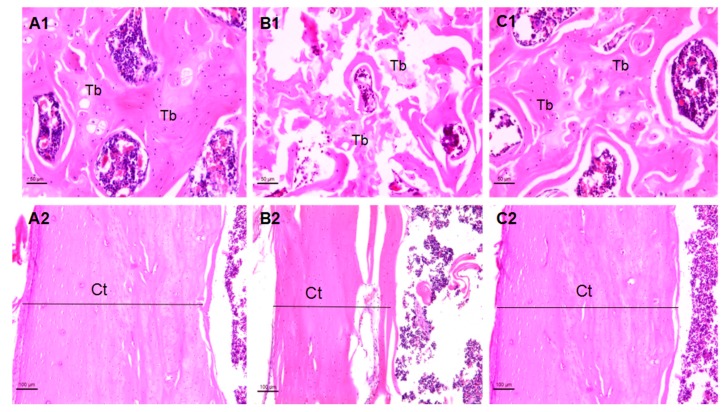
The femoral and tibia morphology of rats in each group. (**A1**) The femur metaphysis in a rat of control group; (**B1**) the femur metaphysis in a rat of T1DM (diabetic) group; (**C1**) the femur metaphysis in a rat of the T1DM-icariin (ICA) group; (**A2**) the tibia in a rat of control group; (**B2**) the tibia in a rat of T1DM group; (**C2**) the tibia in a rat of the T1DM-ICA group. Hematoxylin and eosin staining, magnification: 200× Tb: Trabecular bone. Ct: Cortical bone.

Histopathological observation showed that diabetes caused morphological changes in femoral trabecular and tibial cortical thickness. As shown in Figure 3(A1–C1), the femoral trabecular spacing of the T1DM group was increased and the femoral trabeculae was broken (Figure 3(B1)); the femoral bone structure in the T1DM-ICA group became normal (Figure 3(C1)). The tibia cortical thickness (Ct.T) was decreased in the T1DM group (Figure 3(B2)), and it was restored in ICA treated group (Figure 3(C2)).

**Figure 4:**
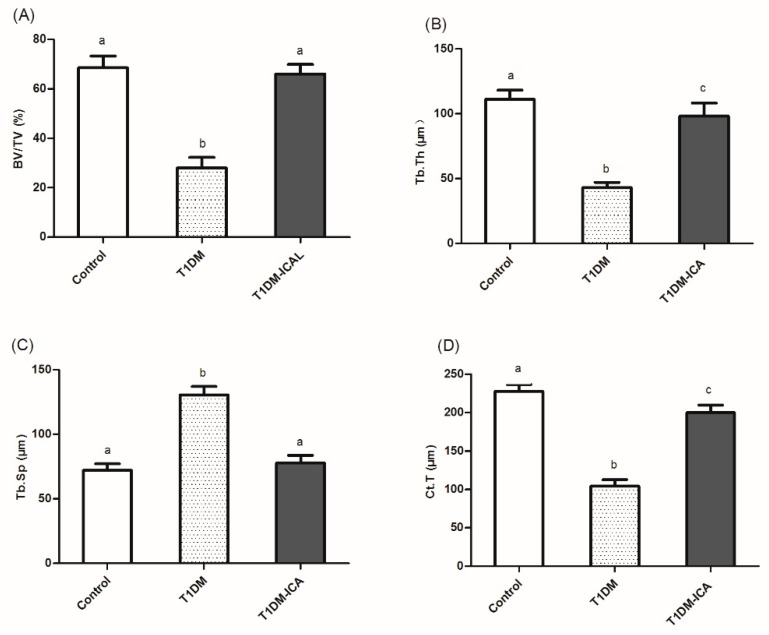
Bone histomorphometric parameters in all experimental groups. (**A**) Bone volume per tissue volume (BV/TV, %); (**B**) trabecular thickness (Tb.Th, μm); (**C**) trabecular separation (Tb.Sp, μm); (**D**) cortical thickness (Ct.T, μm). Values are presented as means ± SD. Different letters indicate statistically significant difference (*p* < 0.05).

As Figure 4 indicates, after 8 weeks of ICA administration the bone histomorphometric parameters became normal in T1DM rats. The bone volume per tissue volume (BV/TV, Figure 4A), trabecular thickness (Tb.Th, Figure 4B), and cortical thickness (Ct.T, Figure 4D) were increased in T1DM-ICA group compared with T1DM group (*p* < 0.01); the trabecular separation (Tb.Sp, Figure 4C) was decreased in T1DM-ICA group compared with T1DM group (*p* < 0.01). These results suggest that ICA can repair diabetes-induced bone structure disorder in rats.

**Figure 5:**
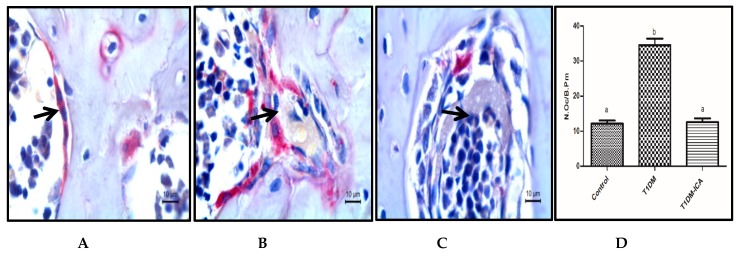
The number of osteoclasts per bone perimeter (N.Oc/B.Pm) in each group. (**A**) Osteoclasts in femur bone tissue of control group rat; (**B**) osteoclast in femur bone tissue of T1DM group rat; (**C**) osteoclast in femur bone tissue of T1DM-ICA group rat; (**D**) the number of osteoclasts per bone perimeter (N.Oc/B.Pm); Values are presented as means ± SD. Different letters indicate statistically significant difference (*p* < 0.05). Femur bone tissue slides were stained by tartrate resistant acid phosphatase (TRAP), the black arrow points to osteoclasts.

As shown in Figure 5, osteoclast was a large multinucleated cell with purplish red color after TRAP staining. Compared with the control group, the number of osteoclasts per bone perimeter (N.Oc/B.Pm) was increased in diabetic rats (T1DM group) (*p* < 0.05), and was significantly decreased after 8 weeks ICA treatment; there was a significant difference in N.Oc/B.Pm between T1DM and T1DM-ICA groups (*p* < 0.05) (Figure 5D).

### 2.5. ICA Decreased Bone Marrow Adipocyte Density and Adipocyte Diameter

**Figure 6:**
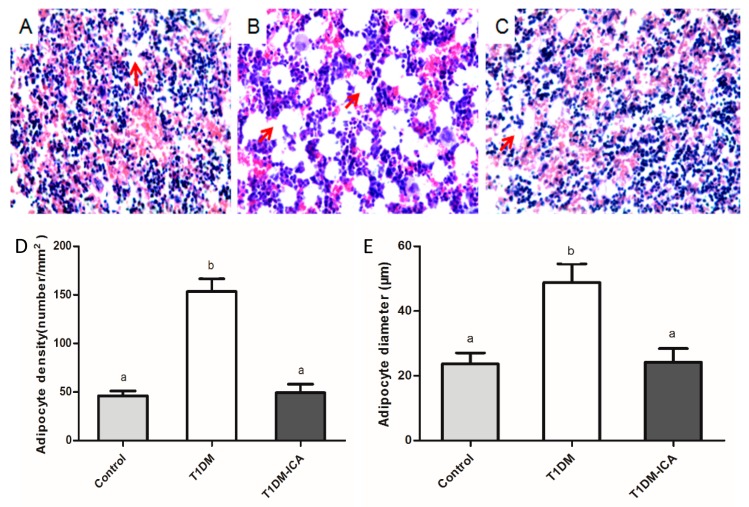
Bone marrow adipocyte and their density and diameter in all experimental groups. (**A**) The tiba bone marrow of control rat; (**B**) the tiba bone marrow of T1DM rat; (**C**) the tiba bone marrow of T1DM-ICA rat; hematoxylin and eosin staining, magnification: 400×; (**D**) adipocyte density of tibia bone marrow in each group; (**E**) mean adipocyte diameter of tibia bone marrow in each group. Values are presented as means ± SD. Different letters indicate statistically significant difference (*p* < 0.05). Red arrows point to adipocytes.

As shown in Figure 6, the number of adipocytes in the bone marrow of T1DM rats (Figure 6B) was higher than that of the control group (Figure 6A), while it was significantly decreased in the T1DM-ICA group (Figure 6C). As Figure 6D,E indicated, the mean adipocyte diameter (μm) and bone marrow adipocyte density of the T1DM group were increased significantly compared with the control (*p* < 0.01); after 8 weeks of ICA administration the bone marrow adipocyte density and adipocyte diameter were decreased significantly in T1DM-ICA group compared with T1DM group (*p* < 0.01).

### 2.6. ICA Increased RUNX 2, OPG mRNA Expression, and OPG/RANKL mRNA Ratio in Bone Tissues of Diabetic Rats

**Figure 7:**
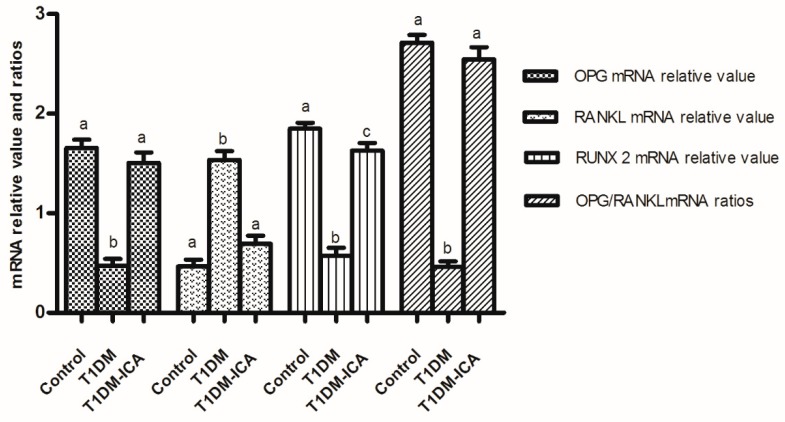
The expression of OPG, RANKL and RUNX 2 mRNA, and OPG/RANKL mRNA ratio in bone tissues of all experimental groups. Values are presented as means ± SD. Different letters (a, b, c) indicate statistically significant difference (*p* < 0.05).

As shown in Figure 7, compared with the control group, the RUNX 2 and OPG mRNA were decreased, RANKL mRNA was increased, and the ratio of OPG/RANKL mRNA was decreased in the T1DM group (*p* < 0.01); after 8 weeks of ICA administration the RUNX 2 and OPG mRNA were increased, RANKL mRNA was decreased, and the ratio of OPG/RANKL mRNA was increased in T1DM-ICA group compared with T1DM group (*p* < 0.01).

### 2.7. ICA Increased RUNX 2, OPG and Decreased RANKL Protein Expression in Bone Tissues of Diabetic Rats

**Figure 8:**
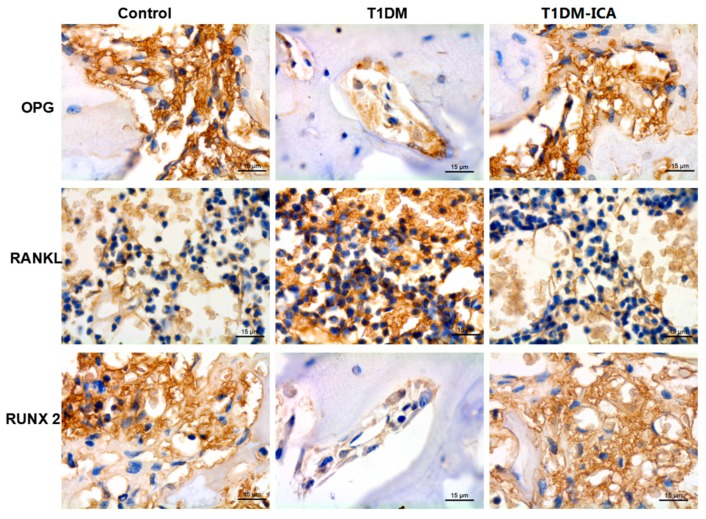
OPG, RANKL, and RUNX 2 protein expression in the femoral bone tissues of each group. The immunohistochemical staining, the cells with positive expression of OPG, RANKL, and RUNX 2 are shown in brown. Magnification: 400x.

**Figure 9:**
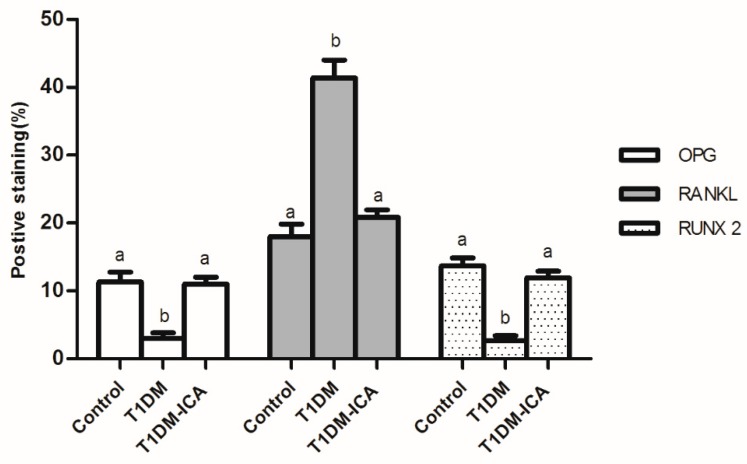
Percentage (%) of the positive staining area of OPG, RANKL, and RUNX 2 in the femoral bone tissues of each group. Values are presented as means ± SD. Different letters (a and b) indicate statistically significant difference (*p* < 0.05).

As shown in Figure 8, compared with the control group, OPG and RUNX2 expression were decreased and RANKL expression was increased in T1DM group. 8 weeks of ICA administration effectively increased bone OPG and RUNX 2 expression in T1DM rats, and decreased bone RANKL expression. As shown in Figure 9, there were significant differences of the positive staining area of OPG, RUNX 2, and RANKL between T1DM-ICA and T1DM group (*p* < 0.05), indicating that ICA supplementation could effectively reduce RANKL and increase the levels of OPG and RUNX 2 in the bone tissue of diabetic rats.

## 3. Discussion

Microstructural changes in diabetic bone disease are characterized by reduced bone mineral density, resulting in bone fragility and increased risk of fracture [20]. Many studies have shown that diabetes affect bone turnover and bone integrity [21,22,23], and the bone loss of diabetes patients is increased and the bone turnover is accelerated [24]. High blood glucose levels affect osteoblast differentiation, impair bone formation, inhibit bone mineralization [25], and induce osteoblast apoptosis, which is considered to be an important reason in diabetic osteopenia [26,27]. Icariin was reported to have bone-protective effects on ovariectomized rats [28,29]. However, whether icariin has protective effects on diabetes-induced osteoporosis has not been reported. In this study, our results indicated that 8-weeks icariin treatment can improve bone loss in diabetic rats.

In this study, T1DM rats showed decreased BMD, increased blood glucose, increased bone turnover markers, increased number of osteoclasts, increased bone marrow adipocyte density, as well as the destruction of bone structure, indicating the DOP rat model was successfully constructed. These indicators were significantly improved after 8 weeks of treatment with ICA, suggesting that ICA has a protective effect on bone loss induced by diabetes in rats.

Hyperglycemia can cause osmotic diuresis; during this study we found that the urinary volume of diabetic rats was increased; the increased urinary volume can cause the excretion of calcium and phosphorus and decrease the calcium concentration in the blood; thus, activated osteoclast promoted the mobilization of bone calcium and phosphorus, enhanced bone resorption, and decreased bone mass [30]. At the same time, sustained hyperglycemia can inhibit the proliferation of osteoblasts and promote osteoclast differentiation, and it is currently believed that high concentration of glucose in the bone marrow microenvironment can cause increased osteoclast differentiation, which may be a pathogenesis of diabetic osteoporosis [31]. In this study, after 8 weeks ICA administration, the blood glucose was significantly decreased in T1DM-ICA group compared with the T1DM group (*p* < 0.01); the hypoglycemic effect of ICA is one of the important reasons for its anti-diabetic-induced osteoporosis.

Bone turnover biomarkers (BTMs) play important roles in the diagnosis and treatment of osteoporosis; they are an important basis for assessing the therapeutic effects of osteoporosis, which reflects bone formation and absorption [32]. BTM includes bone resorption and formation markers [33]. Osteoblasts synthesize osteogenic markers, such as osteocalcin, ALP, and PINP, which reflect the osteogenic function of the body [34]. CTX-1 and TRACP 5b are markers of bone resorption [35,36,37], the higher the levels of TRACP 5b and CTX-1, the lower the bone mineral density [38,39,40]. In this study, both the markers of bone formation and bone resorption were increased in diabetic rats, indicating that the bone turnover was increased. The main reason for the increase of serum bone formation markers in diabetic rats may be that the osteoblasts try to compensate for the bone loss caused by type 1 diabetes. These BTM were normalized in the T1DM-ICA group, suggesting that oral administration of ICA can increase bone mass by inhibiting bone turnover.

In addition to bone mineral density, bone loss can also be demonstrated by bone structure parameters such as decreased bone volume, increased trabecular bone separation, etc. [41]. This study found that the bone structure of diabetic rats was destroyed and bone mineral density was reduced. 8 weeks of ICA treatment can increase bone mineral density, trabecular thickness, and bone volume, decrease the number of osteoclasts and bone marrow adipocyte density, and improve bone structure in diabetic rats, suggesting that ICA has protective effects on bone loss in diabetic rats. It was reported that, in patients with osteoporosis, decreased bone mineral density is associated with increased bone marrow adipocytes; the number of bone marrow adipocytes is an important indicator of osteoporosis [42,43]. In this experiment, we found that the number of bone marrow adipocytes and the average diameter of adipocytes (μm) were increased in diabetic rats, which were restored in ICA treated group. Osteoblasts and adipocytes share a common precursor cell in bone marrow [44], the decreased bone mineral density in diabetic rats can be explained by the tendency of precursor cells to differentiate into adipocytes rather than osteoblasts. ICA treatment can effectively reduce bone marrow lipogenesis in diabetic rats, which is one of the important mechanisms to prevent bone loss caused by diabetes.

RUNX2 is a transcription factor that controls osteoblast differentiation by regulating the gene expression of the extracellular matrix protein [45], and it is one of the genes for the pathogenesis of osteoporosis. In this study, decreased expression of RUNX 2 in serum and bone tissue of diabetic rats suggested that the osteogenic function was affected. However, the restored RUNX2 expression after 8 weeks ICA treatment suggested that ICA could promote osteogenesis by upregulation of RUNX2. OPG/RANKL/RANK signals are related to the occurrence and development of bone metabolic disorders such as osteoporosis. OPG and RANKL are key factors mediating osteoclast differentiation. RANKL can inhibit osteoclast apoptosis and promote osteoclast differentiation [46]. OPG is a soluble receptor secreted by osteoblasts that inhibits the formation of osteoclasts [47,48]. Therefore, the OPG/RANKL concentration ratio is very important for maintaining bone mass and BMD; if this balance is broken, it will lead to the occurrence of metabolic bone diseases, such as osteoporosis, and the reduction of the OPG/RANKL ratio is a manifestation of increased osteolysis [49,50]. In this study, ICA administration for 8 weeks increased the OPG expression and OPG/RANKL expression ratio in diabetic rats, and decreased the level of RANKL in serum and bone tissue. This suggests that ICA can prevent diabetic osteoporosis by up-regulating the expression of RUNX 2 and the OPG/RANKL ratio.

## 4. Materials and Methods 

### 4.1. Animals

8-week-old SD rats (female) weighing 212 ± 14 g were purchased from Chengdu Dashuo Experimental Animal Company (Chengdu, China). The care and operation of the experimental animals were in accordance with the approved plan of the Animal Ethics Committee of Shaanxi University of Technology (Project identification code 2018-054). During the experiment, rats were housed in a room with constant temperature (24 °C) and humidity (50 ± 18%) in individual cages, and the rats were given free access to standard ingredient chow (solid) and distilled water.

### 4.2. Establishment of Rat Model of Diabetes and ICA Administration

After 7 days of adaptive feeding, rats in the model group were intraperitoneally injected with streptozotocin (STZ) (Sigma-Aldrich, St Louis, MO, USA) (60 mg/kg body weight in 100 mL of sterile citrate buffer, pH 4.5) to induce diabetes mellitus, the dosage of STZ was based on our previous study [51]. Rats in the control group were injected with citrate vehicle alone. After 72 h of STZ injection, blood glucose was detected by using an ACCU-CHEK advantage glucometer (Roche Diagnostics, Indianapolis, IN, USA) to confirm the diabetic state. Animals with venous blood glucose levels of over 16.7 mmol/L were considered diabetic and selected for further studies. Then the animals were assigned to three groups (10 rats in each group), they are: (1) Control group (CON); (2) diabetic group (T1DM); and (3) T1DM-ICA group (diabetic rats received 100 mg/kg/d of ICA by intragastric administration for 8 weeks). In T1DM and control groups, rats were given intragastric deionized water instead of ICA. The body weight and blood glucose of each rat was measured at one week intervals during the experimental period, the food intake and water consumption were recorded every day in each group. ICA (purity > 98%) used in this study was purchased from Shanghai Yuanye Bio-Technology (Shanghai, China). The dosage of ICA used in this research was based on the reference of Jing Zhang et al. [52], the ICA was dissolved in deionized water, and the rats were given intragastric ICA every day during the experiment.

### 4.3. Serum Bone Turnover Markers, Ca, P, OPG, RANKL, and RUNX 2 Detection

After 8 weeks of ICA treatment, all rats were fasted for 12 h, and were sacrificed after anaesthesia with excessive isoflurane. Abdominal aorta blood was taken and centrifuged at 4 °C for 15 min to extract serum. Serum calcium (Ca), and phosphorus (P) levels of rats were detected by atomic absorption spectrometer. Serum bone turnover markers, including ALP, osteocalcin (OC), CTX-1, PINP, and TRACP 5b, as well as serum OPG, RANKL, and RUNX 2 were measured according to the protocals of an enzyme-linked immunosorbent assay (Beijing kits Sinogene Bio-Technology Company, Beijing, China).

### 4.4. Bone Mineral Density Measurement

After blood collecting, the bone mineral density (BMD) of the left femur and lumbar vertebrae (L1–L4) of rats was measured using a dual energy X-ray absorptiometry (DEXA) scanning system (Lunar, WI, USA).

### 4.5. Bone Histomorphometric Analysis

Histomorphometric analysis was carried out by the methods previously reported in our laboratory [53]. Right femur and tibia tissues were fixed in 4% paraformaldehyde (PFA) solution then decalcified in 10% EDTA (pH 8.0) at 4 °C for 28 days [54]. Bone tissues were dehydrated in 75%, 85%, 90%, 95%, and 100% ethanol solutions, transparented with xylene, and then embedded in paraffin. 5 µm sections were prepared for histological analyses, and the slides were histologically examined with hematoxylin and eosin staining under Leica DM 3000 microscope (Leica Microsystems, Wetzlar, Germany). Image Pro Plus 5.0 analytic software (Media Cybernetics, Baltimore, MD, USA) was used to measure cortical or trabecular thickness (Ct.T, μm; Tb.Th, μm), trabecular separation (Tb.Sp, μm), and bone volume per tissue volume (BV/TV, %). Acid Phosphatase Kit (Jiancheng Bio-Technology Company, Beijing, China) was used to perform tartrate resistant acid phosphatase (TRAP) staining of femur slides, and the number of osteoclasts was quantified using Image Pro Plus 5.0 analytic software [55].

### 4.6. Bone Marrow Adipocyte Parameters Analysis

Tibial sections stained with hematoxylin and eosin were observed in Leica DM 3000 (Leica Microsystems, Wetzlar, Germany). According to published methods [56], the mean adipocyte diameter (μm) and adipocyte count (mm^2^) in the tibial bone marrow were analyzed using Image Pro Plus 5.0 analysis software.

### 4.7. Immunohistochemistry 

The femur slides (5 μm thick) were incubated with 1% Triton X-100 solution at room temperature for 30 min, then soaked in citric acid buffer solution for 12 min in the microwave oven. Sections were washed three times with PBS-T and then blocked with 3% bovine serum albumin. Primary antibody of RUNX 2, OPG, and RANKL (Invitrogen, Carlsbad, CA, USA) were added to the femoral slides, and incubated at 37 °C 1.5 h, then horseradish peroxidase (HRP) secondary antibodies (1:250) was added, and incubated at 37 °C for 2 h, and the glass slide was washed with PBS-T three times. Then the DAB solution was added to the slide and the nuclei were restained with hematoxylin. Images were observed using a Leica DM 3000 microscope (Leica Microsystems, Wetzlar, Germany). Finally, the percentage of RUNX2, OPG, and RANKL-positive regions was quantitatively analyzed using Image Pro Plus 5.0 analysis software.

### 4.8. Quantitative Real-Time PCR

RUNX 2, OPG, and RANKL gene expression were detected by real-time quantitative PCR. Total RNA from bone tissue was extracted using RNA TRIzol reagent (Sigma-Aldrich, Steinheim am Albuch, Germany). The cDNA was obtained with PrimeScript™ RT Master Mix (TaKaRa, Japan). Real-time quantitative PCR analysis of the gene expression levels of RUNX 2, OPG, and RANKL using the primer sequences listed in Table 3. The gene relative variation expression was analyzed by 2^-ΔΔCT^ method.

### 4.9. Statistical Analysis

All results are presented as the mean ± SD. The data between each group were analyzed using SPSS version 18.0 one-way ANOVA and Duncan’s test. Differences were considered significant when *p* < 0.05.

## 5. Conclusions

The present study indicates that oral administration of ICA has protective effects on diabetic-induced osteoporosis. This was demonstrated by increased BMD, decreased bone turnover markers, decreased bone marrow adipogenesis, increased OPG/RANKL ratio, increased RUNX 2 expression, improved bone architecture, etc. This study demonstrates that the protective mechanism of ICA on diabetes-induced bone loss was that ICA can reduce blood glucose, inhibit bone turnover, suppress bone marrow lipogenesis, and up-regulate OPG/RANKL ratio and RUNX 2 expression. Thus, the present results suggest that ICA may be a potential drug or functional food for treating osteoporosis in diabetic patient.

## Figures and Tables

**Table 1:** Blood glucose, and serum bone turnover markers in each experimental group.

Parameter	Control	T1DM	T1DM-ICA
Glucose (mg/dL)	88.56 ± 7.41 ^a^	417.34 ± 29.64 ^b^	98.45 ± 9.04 ^a^
ALP (U/dL)	104.31 ±10.91 ^a^	200.56 ± 18.59 ^b^	118.78 ± 11.78 ^a^
CTX-1 (ng/mL)	24.31 ± 4.07 ^a^	107.96 ± 13.67 ^b^	30.56 ± 4.16 ^a^
Osteocalcin (ng/mL)	17.39 ± 2.91 ^a^	43.16 ± 6.55 ^b^	25.76 ± 4.18 ^c^
TRACP 5b (U/L)	1.79 ± 0.33 ^a^	3.90 ± 0.72 ^b^	2.21 ± 0.43 ^a^
PINP (μg/L)	44.78 ± 6.01 ^a^	70.84 ± 7.89 ^b^	46.90 ± 6.01 ^a^

Values are presented by mean ± SD. Different letters within rows are used to indicate statistically significant difference (*p* < 0.01).

**Table 2:** Serum calcium (Ca), phosphorus (P), osteoprotegerin (OPG), receptor activator of nuclear factor-κ B ligand (RANKL), and runt-related transcription factor 2 (RUNX 2) in each experimental group.

Parameter	Control	T1DM	T1DM-ICA
Ca (mg/dL)	9.49 ± 0.82 ^a^	4.74 ± 0.63 ^b^	9.45 ± 0.70 ^a^
P (mg/dL)	7.69 ± 0.45 ^a^	3.89 ± 0.65 ^b^	5.89 ± 0.56 ^c^
RUNX 2 (ng/mL)	10.96 ± 2.18 ^a^	3.18 ± 0.54 ^b^	9.64 ± 1.96 ^a^
OPG (ng/mL)	8.79 ± 2.54 ^a^	2.17 ± 0.61 ^b^	8.42 ± 1.35 ^a^
RANKL (ng/mL)	2.33 ± 0.46 ^a^	7.49 ± 1.21 ^b^	2.49 ± 0.38 ^a^
OPG/RANKL ratio	4.21 ± 0.51 ^a^	0.54 ± 0.10 ^b^	3.78 ± 0.46 ^a^

Values are presented by mean ± SD. Different letters within rows are used to indicate statistically significant difference (*p* < 0.05).

**Table 3:** qPCR primer sequences.

Primer Name	Primer Sequence (5–3′)
β-actin-F	GAG ACC TTC AAC ACC CCA GCC
β-actin-R	GGC CAT CTC TTG CTC GAA GTC
RUNX 2-F	CGA AAT GCC TCT GCT GTT AT
RUNX 2-R	TTC TGT CTG TGC CTT CTT GG
OPG-F	ATG TAC GCA CTC AAG CAC TT
OPG-R	AAA GAG TTT CTG ATA CAA TCG GTA C
RANKL-F	TTT CAA GGG GCC GTG CAA AG
RANKL-R	AGC CAC GAA CCT TCC ATC ATA
